# An Ultra-Thin Screen-Printed Conductive Film for Fragment Velocity Measurement: Design, Simulation, and Experimental Validation

**DOI:** 10.3390/s26185792

**Published:** 2026-09-12

**Authors:** Wanchun Jiang, Jun Yang, Jin Li, Difeng Sun, Min Zhang, Ze Jiao, WenXiang Liu, Dezhi Zhang

**Affiliations:** National Key Laboratory of Intense Pulsed Radiation Simulation and Effect, Northwest Institute of Nuclear Technology, Xi’an 710024, China; yangjun@nint.ac.cn (J.Y.); lijin@nint.ac.cn (J.L.); sundifeng@nint.ac.cn (D.S.); zhangmin@nint.ac.cn (M.Z.); jiaoze@nint.ac.cn (Z.J.); liuwenxian@nint.ac.cn (W.L.); zhangdezh@nint.ac.cn (D.Z.)

**Keywords:** fragment velocity measurement, velocity measurement film, response time, kinetic energy loss

## Abstract

Laser-screen targets suffer from prolonged response time, and printed-circuit-board (PCB) targets induce substantial kinetic energy loss of fragments upon penetration. To mitigate these technical limitations, this study develops a novel screen-printed velocity measurement film (VMF) characterized by a fast response time and low kinetic energy loss. The proposed VMF consists of silver conductive grids and a polyethylene terephthalate (PET) substrate, with an ultra-thin overall thickness of 0.175 mm and identical width and spacing of 0.8 mm for all silver strips. This work comprehensively elaborates the film fabrication procedure and validates its practical engineering performance through live-fire fragment penetration experiments. Experimental results demonstrate that the VMF achieves an average response time of 0.14 μs under metallic fragment impact, with average fragment kinetic energy losses of 1.32 J and 2.09 J when penetrating the first and second VMF layers, respectively. Owing to its rapid transient response and minimal interference with fragment flight trajectories, the designed VMF effectively improves the precision of fragment velocity measurement, possessing great application potential for field blast-testing scenarios.

## 1. Introduction

Accurate acquisition of fragment velocity is critical for evaluating warhead damage effectiveness and analyzing dynamic flight characteristics of explosive fragments [1,2,3]. Existing fragment velocity testing techniques are generally classified into non-contact and contact measurement categories. Response time of fragment velocity measurement target refers to the rise time of the electrical signal. It is defined as the time taken by the velocity measurement target from fragment detection to the output of a recognizable electrical signal.

Typical non-contact measurement approaches include X-ray imaging, high-speed photography, radar detection, acoustic target testing, and laser-screen targeting. X-ray imaging enables fragment trajectory visualization despite the interference of flame and smoke, yet it is restricted to small-scale static blast tests due to complex system configuration and limited detection coverage [4]. High-speed photography reconstructs fragment motion trajectories based on binocular vision principles, while its practical application is constrained by a narrow field of view, cumbersome calibration procedures, and stringent protective requirements [5,6]. Doppler radar captures fragment motion parameters by detecting electromagnetic wave frequency shifts, but its bulky structure severely limits field deployment flexibility. Acoustic targets identify fragment arrival time according to generated shock wave signals, whereas they fail to detect subsonic fragments [7,8]. The laser screen target method measures fragment velocity based on the on–off state of the optical path, representing a widely adopted non-contact fragment measurement technique. However, due to challenges in protection, the laser-screen target is only applicable to velocity measurement of fragments along fixed ballistic trajectories in laboratory settings. Moreover, the fragment passing-target waveforms acquired by the laser-screen target suffer from long rising edges and severe background noise [9,10,11].

Contact measurement methods acquire fragment velocity by capturing switching electrical signals generated during fragment penetration, featuring simple structural configuration and stable operational performance, despite relatively complex power supply and signal transmission systems. Common contact sensing targets include metal foil, resistor arrays, PVDF films, and printed-circuit-board (PCB) targets [12,13,14]. Metal foil targets realize velocity measurement through conductive switching of double-layer metal structures. A three-layer metal foil projectile sensor proposed by Chinke et al. [15] achieves a velocity measurement accuracy of 98% compared with Doppler radar systems. Shao et al. [16] developed a five-layer metal foil connected–disconnected probe, consisting of double metal layers, an insulating layer, and dual sealing layers, for underwater jet velocity testing of warheads. Yin et al. designed a variable-impedance resistor target with 80 impedance loops and an initial resistance of 200 Ω, which can identify multiple fragments via stepped waveform signals. PVDF films detect fragment arrival time by capturing piezoelectric charge variations, and the PVDF-based testing system proposed by Wang et al. can evaluate weapon damage areas based on fragment penetration distribution [17,18]. PCB targets calculate fragment arrival time through copper foil conduction continuity, with the advantages of low cost and convenient deployment. However, conventional PCB targets adopt copper conductive layers and thick glass fiber substrates (thickness > 2 mm), which cause substantial fragment kinetic energy loss, ultimately restricting measurement precision in static blast tests [18].

To address the slow response of laser-screen targets and the high kinetic energy loss of PCB targets, this study proposes a novel screen-printed velocity measurement film (VMF) with a fast response time and low motion interference. The sensor adopts high-conductivity silver grids with uniform strip width and spacing of 0.8 mm, paired with an ultra-thin 0.175 mm PET substrate. This optimized structural design guarantees a rapid response to small-diameter fragment impact and effectively suppresses fragment kinetic energy loss during penetration. The VMF designed in this work not only addresses the problems of long response time and severe background noise existing in laser-screen targets but also resolves the excessive kinetic energy loss of PCB targets. Benefiting from its large sensing area and small spacing between metal strips, the film can respond to fragments with smaller diameters over a wide range and thus possesses promising application prospects.

## 2. Materials and Methods

**Figure 1:**
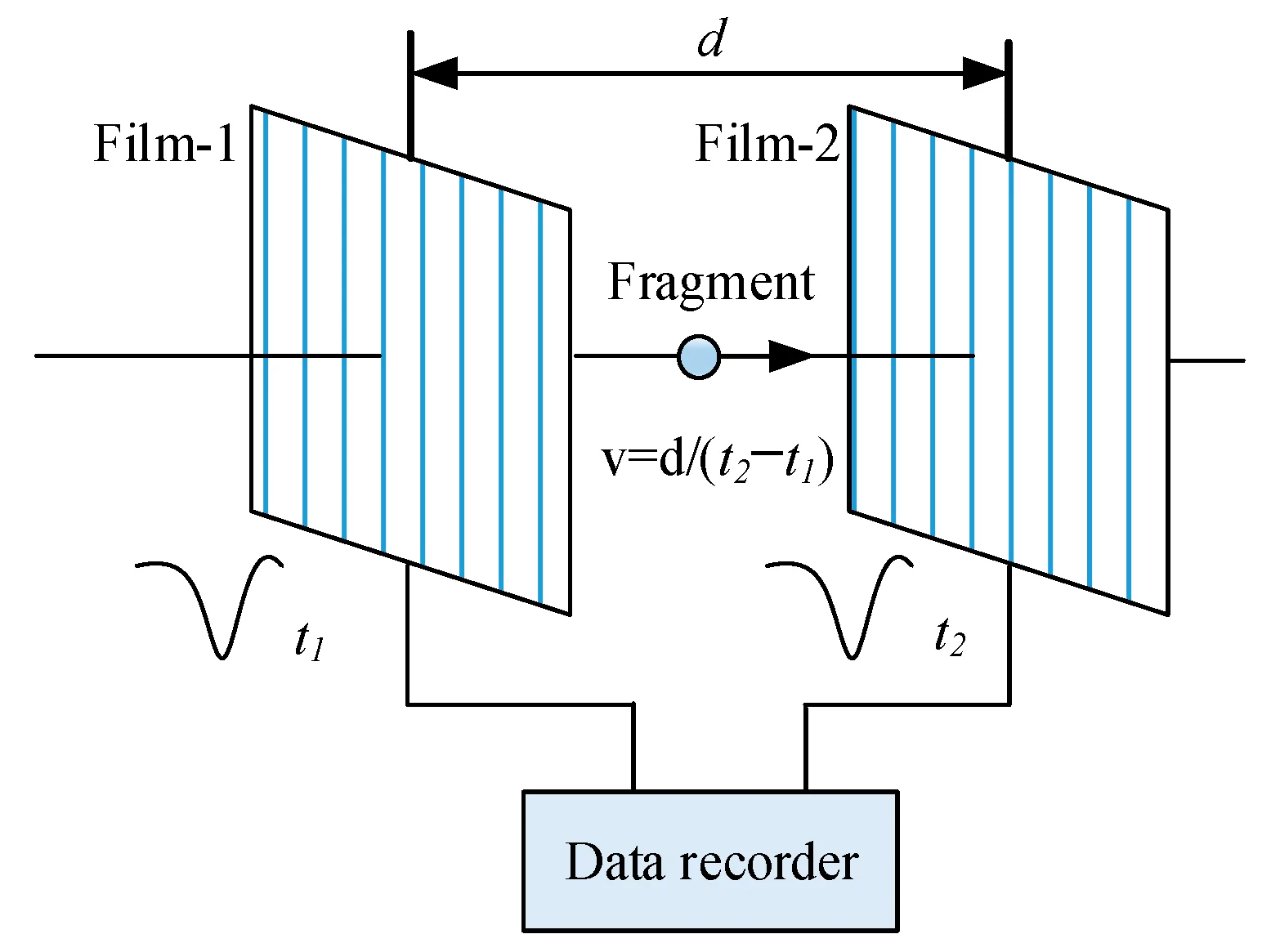
The working principle of the proposed VMF.

The basic working principle of the proposed VMF is based on the conductive switching effect, as illustrated in Figure 1. When a flying fragment impacts and penetrates the film, electrical conduction is established between two mutually isolated metal grid groups, inducing an abrupt voltage mutation. A high-speed data acquisition system records the transient voltage signal, and fragment velocity is calculated by extracting the time node of voltage variation, with the voltage-variation duration defined as the film response time. The response performance of the VMF is dominated by grid conductivity and substrate thickness: higher grid conductivity shortens the signal response time, while thinner substrates reduce fragment kinetic energy dissipation. In contrast, conventional PCB-based velocity measurement films adopt copper grids and thick glass fiber epoxy substrates, resulting in excessive thickness (approximately 2 mm). Such structural limitations lead to severe kinetic energy loss and delayed signal response, significantly degrading velocity measurement accuracy.

### 2.1. Structural Design and Fabrication Process

**Figure 2:**
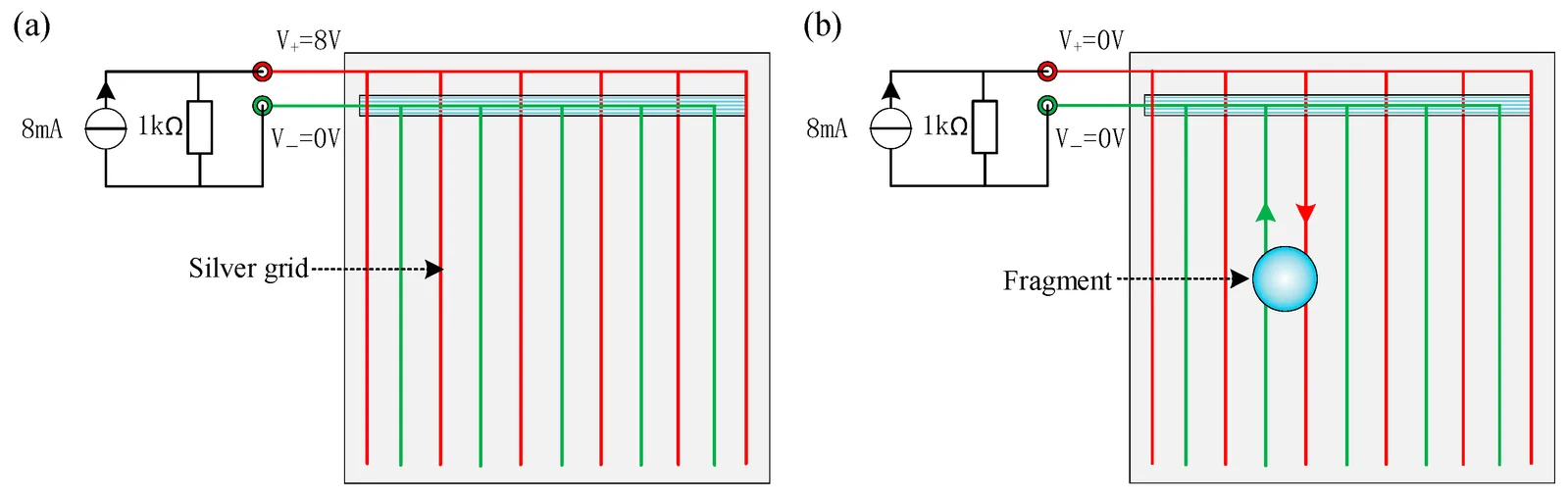
Structure of silk-screen film: (**a**) dual grids insulated; (**b**) dual grids short-circuited.

To overcome the low conductivity and excessive thickness drawbacks of traditional velocity measurement films, this study develops a high-performance VMF via screen-printing technology. The core structure consists of two electrically isolated metal grid arrays for fragment penetration signal generation. During testing, a 1 kΩ shunt resistor is connected in parallel between the two grid groups, which are powered by an 8 mA constant current source. Under static conditions, the dual grids remain electrically insulated, maintaining a stable initial voltage of 8 V (8 mA × 1 kΩ), as shown in Figure 2a. Fragment impact induces grid short-circuiting, resulting in an immediate voltage drop to 0 V and generating a characteristic pulse signal for velocity acquisition (Figure 2b).

The integrated VMF structure comprises a PET base substrate, two silver grid arrays, insulating isolation strips, metal pressure fastening rings, and copper signal leads. The effective sensing area of the film reaches 540 mm × 540 mm on a 600 mm × 600 mm PET substrate with a thickness of 0.175 mm. Each silver grid array is composed of 169 vertical silver strips and one parallel horizontal silver strip, all with a uniform width and spacing of 0.8 mm. The two sets of silver grid arrays together include 169 × 2 = 338 silver strips with 337 inter-strip gaps. Therefore, the total width is calculated as 338 × 0.8 mm + 337 × 0.8 mm = 540 mm. The horizontal strip connects all vertical strips in parallel and extends outward to link with external copper leads via metal pressure rings. Insulating strips are embedded at grid overlapping regions to prevent undesired electrical conduction. Considering the fragility of fine silver grids, traditional soldering is abandoned, and mechanical compression connection via metal pressure rings and 1 mm diameter copper leads is adopted to ensure stable electrical signal transmission to the data acquisition instrument.

**Figure 3:**
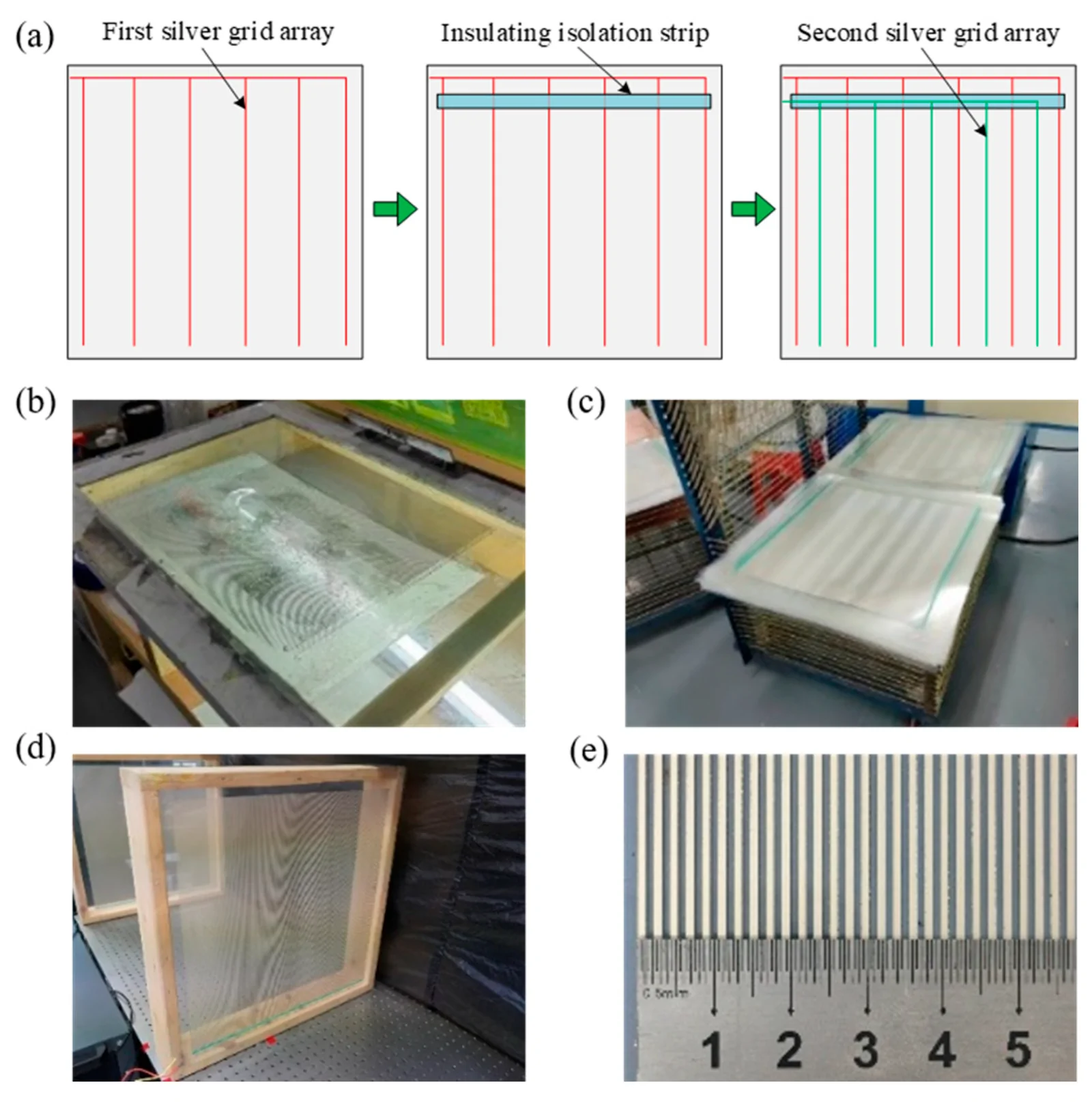
Production process of silk-screen film: (**a**) schematic diagram of fabrication process; (**b**) screen-printing; (**c**) light curing; (**d**,**e**) finished product.

The screen-printing fabrication process of the VMF is standardized, repeatable, and scalable, as presented in Figure 3. First, vector patterns of silver grids and insulating strips are designed using mapping software and imported into a laser typesetter. Infrared laser scanning is performed to prepare three precision-film templates matching the structural layout of double-layer silver grids and single-layer insulating strips. Thereafter, a screen frame is assembled by fixing a silk screen onto an aluminum alloy frame, and a uniform layer of photosensitive glue is coated on the screen surface. Dedicated screen-printing plates are fabricated by laminating the film templates onto the photosensitive screen, followed by ultraviolet exposure, water washing, and drying procedures. Three independent screen-printing plates are manufactured by repeating the above process for the three sets of templates. For film preparation, silver paste and insulating ink are mixed at a fixed ratio, and a polyethylene terephthalate (PET) substrate is firmly fixed on the printing platform. The first screen-printing plate is positioned over the PET substrate for the scraping and deposition of the first silver-paste layer, which is subsequently cured and dried under ultraviolet light. The second plate is then used to print insulating ink, followed by another round of ultraviolet curing and drying. The third plate is applied to deposit the second silver grid layer, and the complete silver grid screen-printed thin film is obtained after final curing and drying. The insulating performance of the dual grid arrays is characterized using a multimeter. The fabricated thin film contains silver strips with a total length of 183 m. The two sets of silver grids exhibit zero electrical conduction and a constant strip spacing of 0.8 mm, demonstrating high fabrication precision and technical difficulty. After successful insulation verification, 20 cm copper leads are assembled via punching to facilitate subsequent experimental testing.

The silver strips of VMF have a width of 0.8 mm, with an identical gap of 0.8 mm between adjacent strips. When a spherical fragment with a diameter of 6 mm makes full contact with the VMF, 6/0.8/2 = 3.75 silver strips are turned on. For conventional PCB targets, the copper strip width is 2 mm, and the gap between adjacent copper strips is also 2 mm. When a 6 mm diameter spherical fragment fully contacts the PCB target, 6/2/2 = 1.75 copper strips are activated. To generate a valid fragment penetration waveform, more than two metal strips need to be turned on. By comparison, it can be readily found that the VMF proposed in this work can not only greatly increase the probability of responding to small-diameter metallic fragments, but also significantly suppress waveform oscillation caused by unstable strip conduction.

### 2.2. Circuit Simulation Setup and Analysis

**Figure 4:**
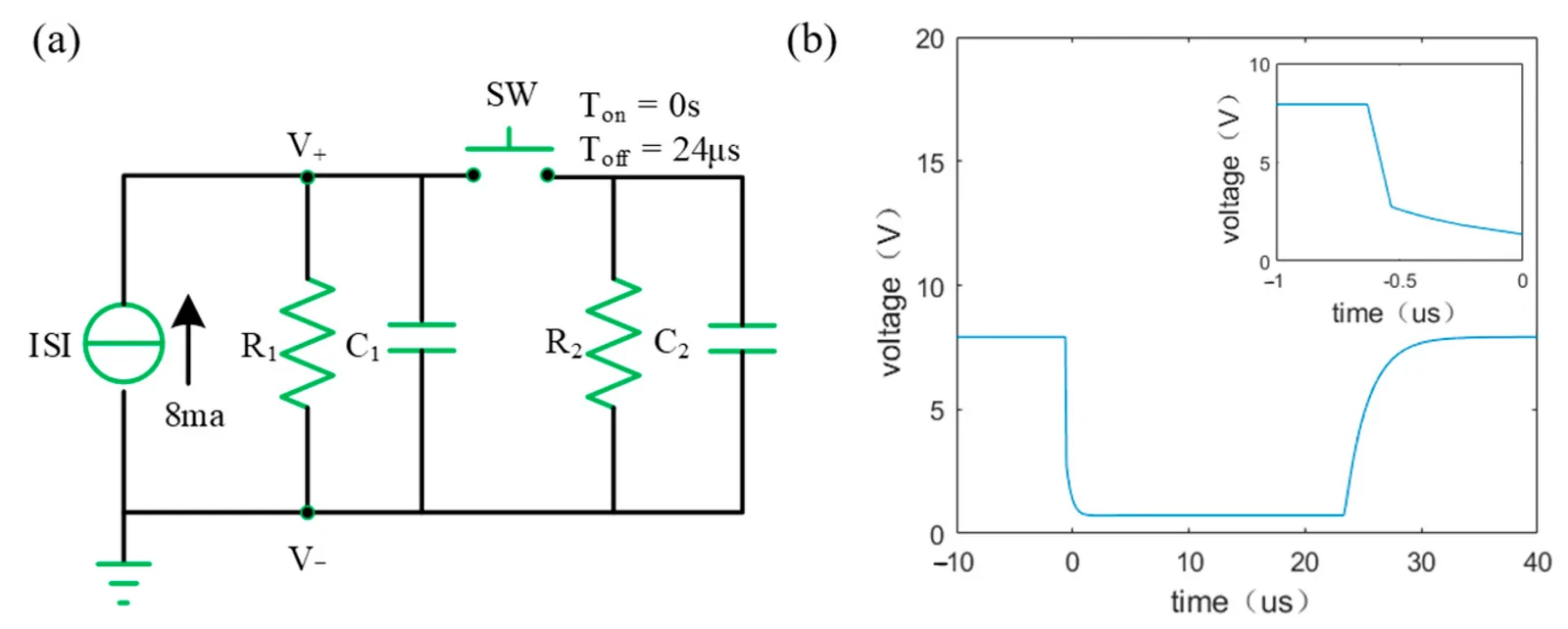
Equivalent circuit model: (**a**) circuit structure; (**b**) circuit simulation.

Based on the electrical configuration of the screen-printed VMF, an equivalent circuit model is established to characterize its transient response to fragment impact (Figure 4a). ISI denotes the IEPE current source with an output current of 8 mA; $R_{1}$ is the parallel-connected resistor with a resistance of 1 kΩ; $C_{1}$ is the parasitic capacitance of $R_{1}$, equal to 2 nF; $R_{2}$ represents the resistance of the metal grid strips, which is 100 Ω; and $C_{2}$ is the parasitic capacitance of $R_{2}$ with a value of 3.1 nF. This set of parameters is acquired by measuring the impedance between the two leads of the film with an impedance analyzer (Impedance analyzer, Model TH2852, Tonghui Electronics Co., Ltd., Changzhou, China). For a 6 mm diameter steel fragment impacting the film at 250 m/s, the effective penetration duration is calibrated as 24.0 μs, which is set as the closing time of the equivalent switch in the simulation model.

Response time ($t_{f}$) is defined as the duration required for an obvious voltage variation to occur in the VMF circuit after fragment impact. Random non-ideal contact conditions are induced by variable fragment impact positions and dynamic collision processes, which prevent the circuit voltage from fully settling at 0 V after electrical shorting. To achieve unified comparative criteria, this work defines response time as the time interval for the voltage to drop to 50% of its initial steady-state magnitude, while the full pulse width ($t_{w}$) stands for the actual fragment penetration duration. The voltage-variation curve of the VMF can be obtained via circuit simulation software, as shown in Figure 4b. Circuit simulation predicts a response time of 0.09 μs and a penetration duration of 25.2 μs for the screen-printed velocity measurement film.

## 3. Experimental Validation

### 3.1. Experimental Platform and Test Configuration

**Figure 5:**
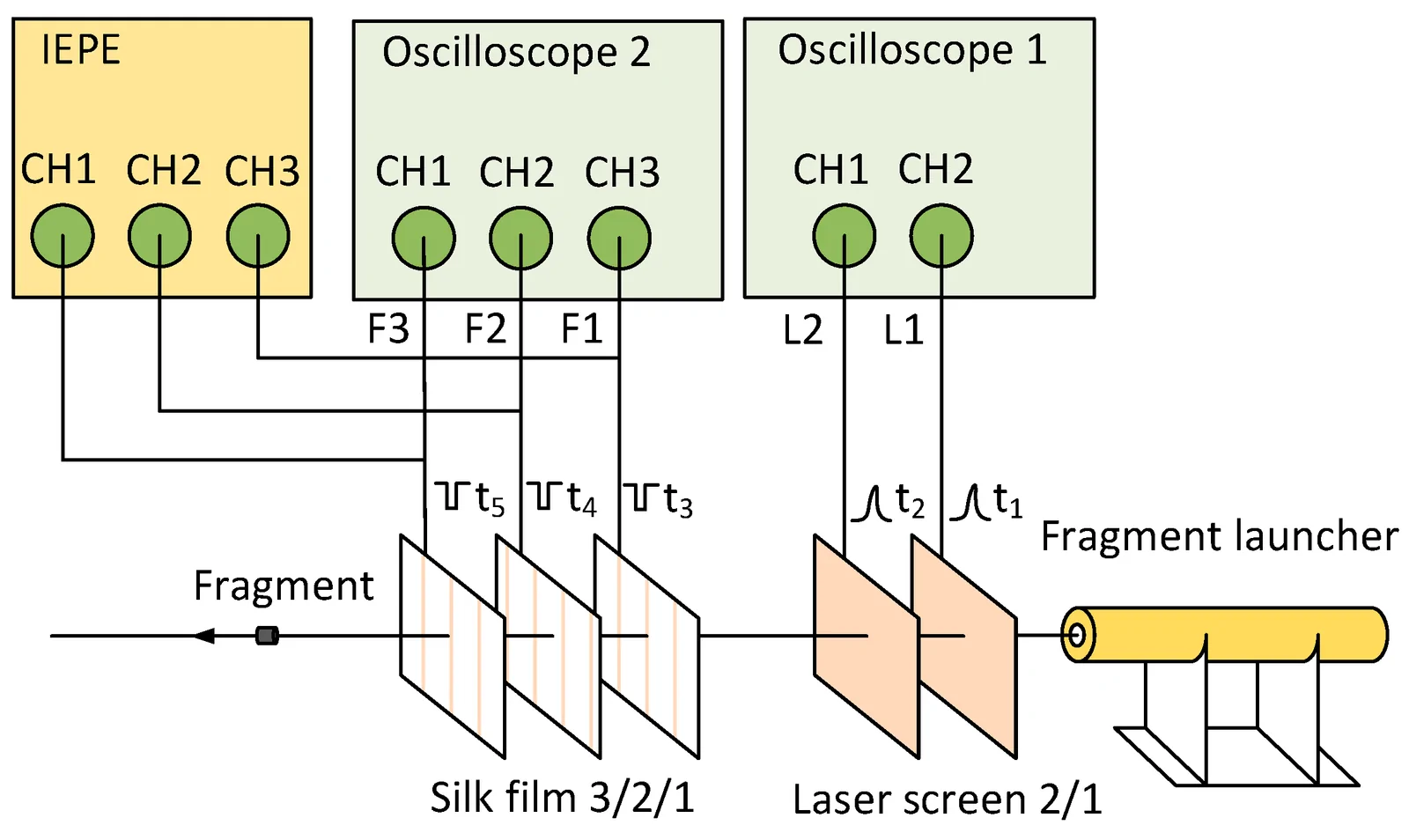
Schematic diagram of the VMF fragment velocity test platform.

A dedicated fragment launch experimental platform is constructed to comprehensively verify the response characteristics and kinetic energy loss performance of the proposed VMF. The experimental system consists of a fragment launcher, dual infrared laser-screen targets, three screen-printed VMFs, an 8 mA constant current source, and two high-speed oscilloscopes (Figure 5). The launcher is capable of accelerating 6 mm diameter stainless steel fragments to a stable flight velocity range of 230–260 m/s. Two infrared laser screens are arranged with a spacing of 200 mm to measure the initial fragment velocity, and the corresponding laser penetration waveforms (L1, L2) are recorded by Oscilloscope 1. The sampling rate of Oscilloscope 2 is 500 MGSa/s, yielding a temporal resolution of 2 ns. Three VMFs are evenly placed at 1 m intervals, and their fragment penetration response waveforms (F1, F2, F3) are captured by Oscilloscope 2. The sampling rate of Oscilloscope 2 is 500 MGSa/s, yielding a temporal resolution of 2 ns. Each VMF is connected in parallel with a 1 kΩ resistor, maintaining a stable initial working voltage of approximately 8 V, which drops to nearly 0 V once fragment penetration occurs. In short, Curve L1 is the waveform generated when the fragment passes through the first laser screen, Curve L2 is the waveform generated when the fragment passes through the second laser screen, Curve F1 is the waveform generated when the fragment passes through the first VMF, Curve F2 is the waveform generated when the fragment passes through the second VMF, and Curve F3 is the waveform generated when the fragment passes through the third VMF.

**Figure 6:**
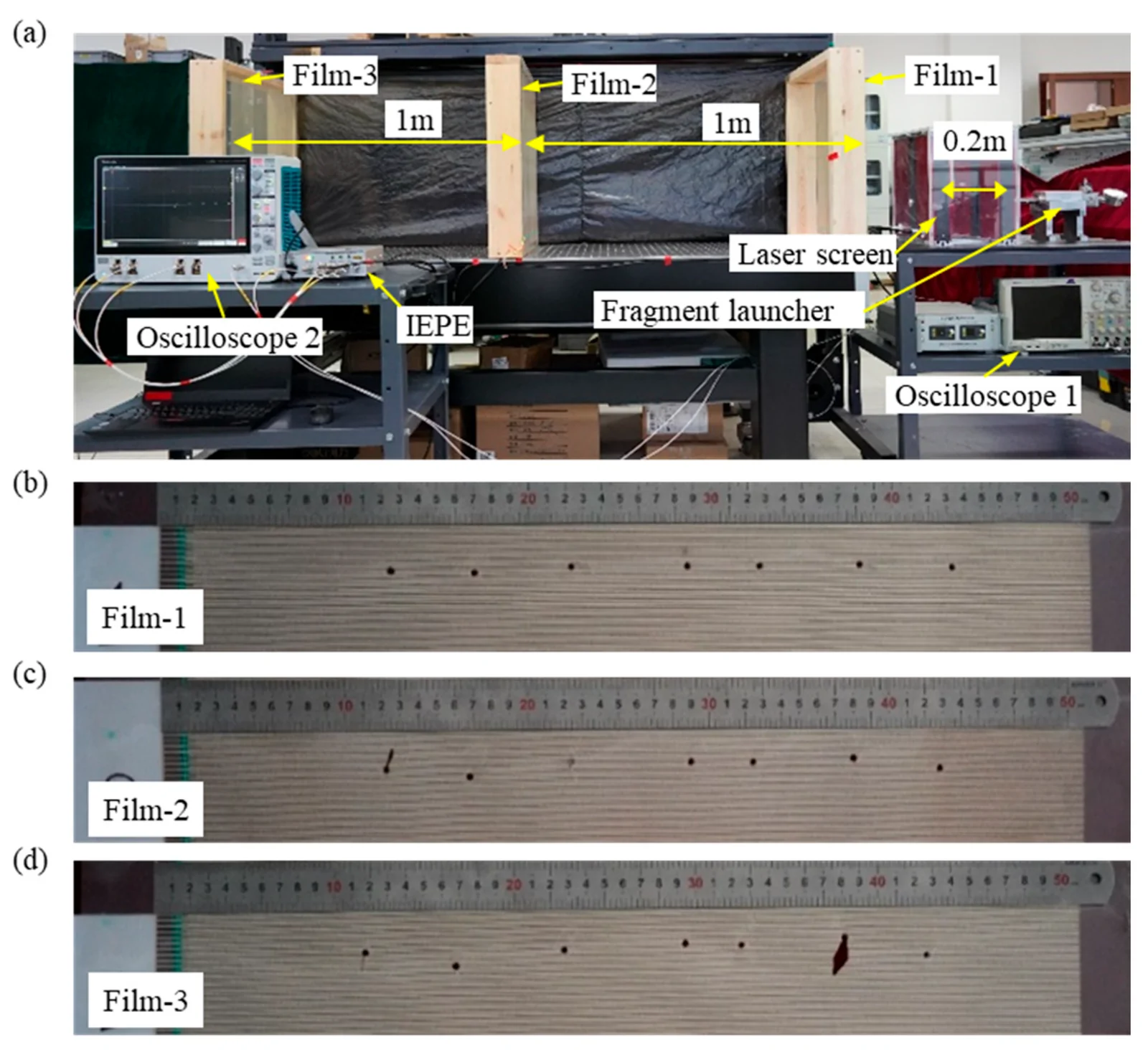
Experimental platform: (**a**) experimental system; (**b**–**d**) fragment holes of film-1, film-2, and film-3.

A total of seven valid fragment launch tests are conducted in this study. The first fragment is used for VMF response time calibration, while the remaining six fragments are adopted for quantitative measurement of film-induced kinetic energy loss. The overall experimental layout and fragment penetration holes on the VMFs are demonstrated in Figure 6.

### 3.2. Transient Response Time Measurement and Analysis

**Figure 7:**
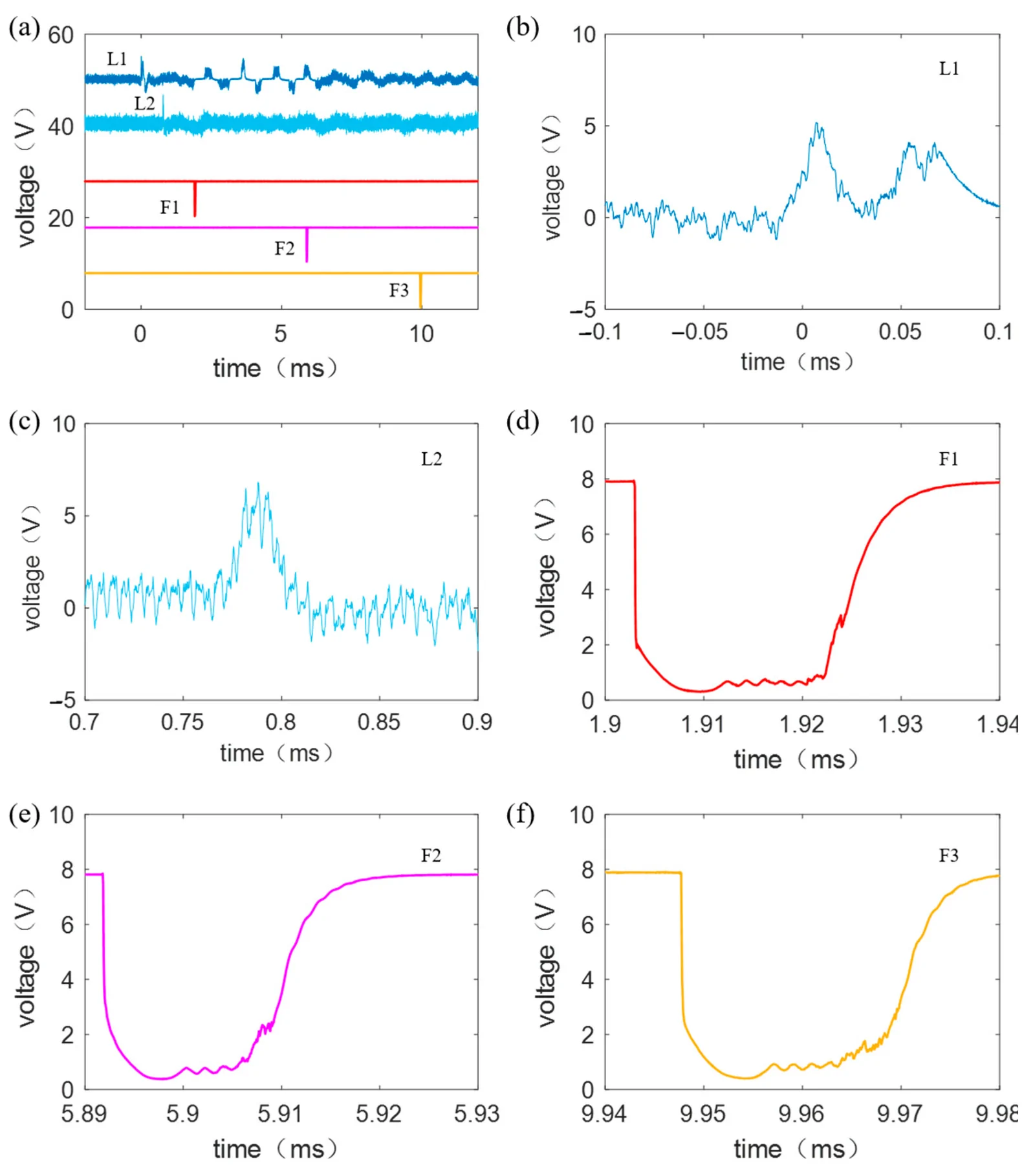
Waveforms of fragment overpasses (1st): (**a**) curves of L1, L2, F1, F2, and F3; (**b**–**f**) magnified image of F1, L2, F1, F2, and F3.

The typical fragment penetration waveforms acquired in the response time test are presented in Figure 7a–f. Signal offsets of 50 V, 40 V, 20 V, 10 V, and 0 V are applied to curves L1, L2, F1, F2, and F3, respectively, to eliminate waveform overlap and facilitate intuitive comparison. The laser screen waveforms (L1, L2) exhibit obvious positive pulses, whose rising edges mark the precise moments of fragment penetration. The rising edges of L1 and L2 occur at 0 μs and 780.3 μs, respectively. Given a fixed laser screen spacing of 200 mm, the calculated initial fragment velocity is 256 m/s, corresponding to a theoretical single-film penetration duration of approximately 24.0 μs. The response time ($t_{f}$) of the laser-screen target is defined as the time taken for the signal amplitude to rise from zero to 50% of its peak value. Local magnification of individual pulse signals yields rising-edge response times of 9.83 μs and 7.81 μs for L1 and L2, with corresponding penetration pulse widths of 12.8 μs and 11.1 μs. The averaged experimental results show that the laser screen achieves an average response time of 8.82 μs and an average penetration pulse width of 12.0 μs, as summarized in Table 1.

All VMF response waveforms (F1, F2, F3) show stable and distinct negative pulse characteristics. In this work, the film response time ($t_{f}$) is defined as the time required for the pulse amplitude to decline by 50% of its initial steady-state value on the falling edge, and the full pulse width ($t_{w}$) represents the actual fragment penetration duration. Local magnification of individual pulse signals yields falling-edge response times of 0.16 μs, 0.12 μs, and 0.13 μs for F1, F2, and F3, with corresponding penetration pulse widths of 19.2 μs, 23.3 μs, and 21.6 μs. The averaged experimental results show that the VMF achieves an average response time of 0.14 μs and an average penetration pulse width of 21.3 μs, as summarized in Table 1.

A comparison reveals that the response time of the VMF is significantly shorter than that of the laser-screen target. Moreover, the waveforms acquired by the VMF are free from the severe background noise observed for the laser-screen target. Furthermore, the pulse width of the VMF is close to 24.0 μs, which indicates that the laser-screen target imposes stricter conditions for responding to fragment passage. Compared with the simulated results (0.09 μs response time and 25.2 μs penetration duration), the VMF experimentally measured response time is slightly longer, while the actual penetration pulse width is marginally shorter. This minor discrepancy is primarily attributed to the randomness of fragment impact positions. When the fragment impacts deviate from the midpoint of two adjacent silver grid strips, the signal conduction path increases, prolonging the response time and shortening the effective grid contact duration, thereby causing consistent deviations between experimental and simulated data.

### 3.3. Kinetic Energy Loss Quantitative Measurement

**Figure 8:**
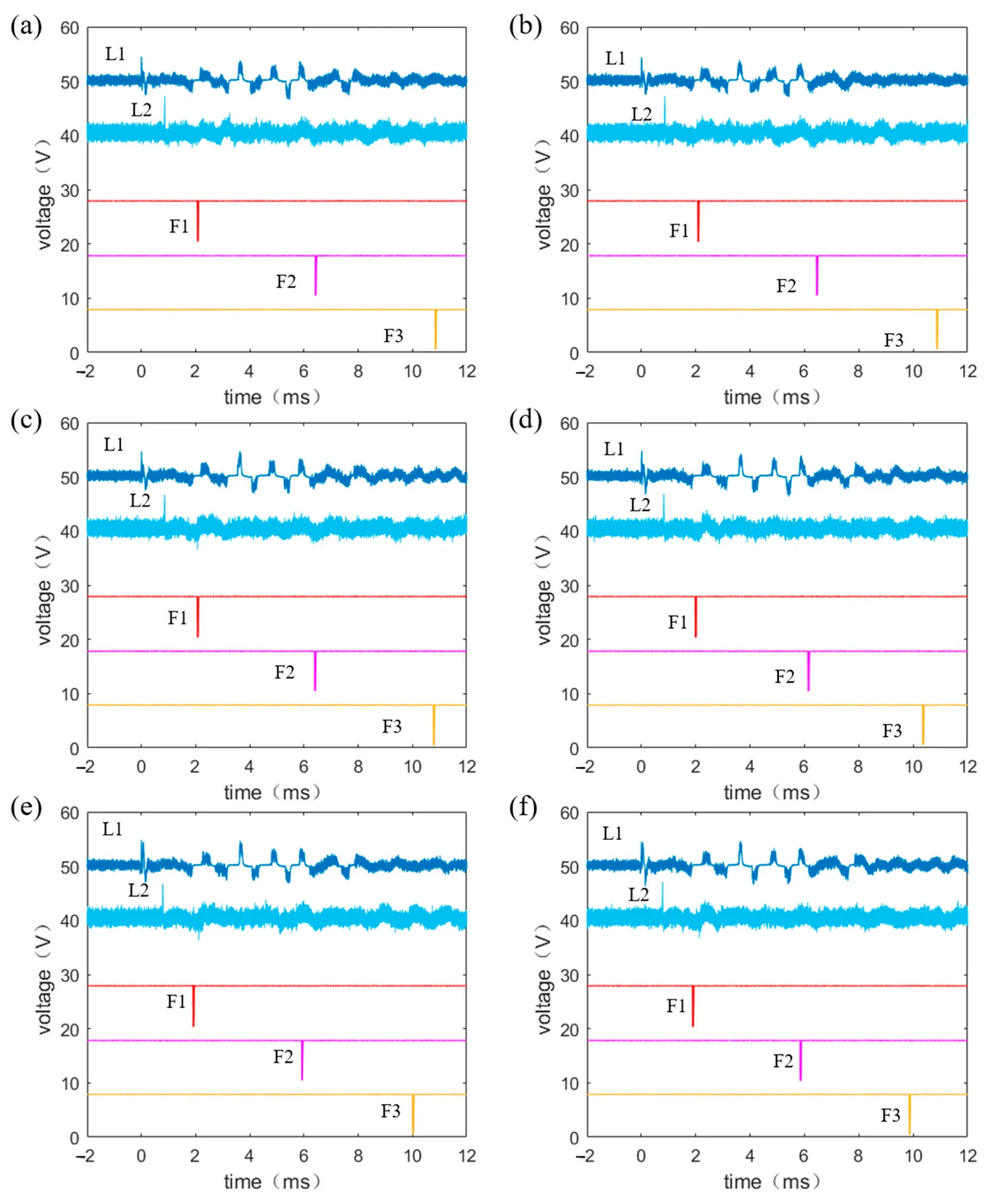
Waveforms of fragment overpasses: (**a**–**f**) 2nd to 7th fragment overpasses.

The typical fragment penetration waveforms acquired in the kinetic energy loss quantitative measurement are presented in Figure 8a–f. Curves L1 and L2 show a distinct positive pulse near time zero, and curves F1, F2, and F3 exhibit a clear negative pulse. The moments corresponding to the rising edges of the two positive pulses ($t_{1},\;t_{2}$) indicate the instants when the fragment arrives at the two laser screens. The moments corresponding to the falling edges of the three negative pulses ($t_{3},\;t_{4},\;t_{5}$) represent the times when the fragment initially contacts the three VMFs, which are defined as the time points when the voltage amplitude drops to half of its initial value, consistent with the aforementioned definition. Therefore, neglecting air resistance, the fragment velocity at each stage can be calculated using the following equation:

(1) $$\left\{\begin{array}{c} v_{0}=\frac{d}{T_{0}}=\frac{d}{t_{2}-t_{1}}\\ v_{1}=\frac{L}{T_{1}}=\frac{L}{t_{4}-t_{3}}\\ v_{2}=\frac{L}{T_{2}}=\frac{L}{t_{5}-t_{4}} \end{array}\right.$$

where $v_{0}$ is the initial velocity of the fragment, $v_{1}$ is the velocity of the fragment after passing through the first VMF, $v_{2}$ is the velocity after passing through the second VMF, d (200 mm) is the distance between the two laser screens, and L (1000 mm) is the distance between two adjacent VMFs. Since $t_{3}$ refers to the instant at which the fragment initially contacts the first VMF and $t_{4}$ marks the moment at which the fragment initially contacts the second VMF, the fragment has not penetrated the second film at$\;t_{4}$. Therefore, $v_{1}$ denotes the fragment velocity after passing through the first VMF. By the same logic, $v_{2}$ stands for the fragment velocity after penetrating the second VMF.

The mass of the fragment is 0.987 g, and the kinetic energy of the fragment at each stage can be calculated according to the following equation:

(2) $$\left\{\begin{array}{c} E_{0}=\frac{1}{2}mv_{0}^{2}\\ E_{1}=\frac{1}{2}mv_{1}^{2}\\ E_{2}=\frac{1}{2}mv_{2}^{2} \end{array}\right.$$

where $E_{0}$ is the initial kinetic energy of the fragment, $E_{1}$ is the kinetic energy after passing through the first VMF, and $E_{2}$ is the kinetic energy after passing through the second VMF. The kinetic energy loss of the fragment at each stage can be calculated using the following equation:

(3) $$\left\{\begin{array}{c} \Delta E_{1}=E_{1}-E_{0}\\ \Delta E_{2}=E_{2}-E_{1} \end{array}\right.$$

where $\Delta E_{1}$ is the kinetic energy loss of the fragment after passing through the first VMF and $\Delta E_{2}$ is the kinetic energy loss after passing through the second VMF.

Statistical results of six valid kinetic energy loss tests are listed in Table 2. The average kinetic energy loss is 1.32 J for the first VMF penetration ($\Delta E_{1}$) and 2.09 J for the second penetration ($\Delta E_{2}$), presenting a consistent trend of $\left|\Delta E_{2}\right|>\left|\Delta E_{1}\right|$. This phenomenon originates from slight fragment flight deflection after the first penetration, which is induced by asymmetric contact force distribution between the fragment and the film. The deflected flight trajectory increases the actual propagation distance between the second and third VMFs, resulting in a lower measured $v_{2}$ value than the real velocity and a corresponding overestimation of secondary kinetic energy loss. The kinetic energy loss is mainly converted into punching fracture energy, vibrational kinetic energy of the film, frictional heat energy, and other forms. Due to the limitations of measurement methods, the specific values of these energy components cannot be accurately determined.

### 3.4. Uncertainty Analysis

The measurement uncertainty of the VMF system follows the JCGM 100:2008 (GUM) standard [19]. Uncertainties are categorized into Type A statistical uncertainty from repeated experiments and Type B systematic uncertainty induced by assembly tolerance, structural deflection, and waveform identification deviation. The fragment flight velocity is performed on Equation (1), where L=1000 mm is the fixed spacing between two VMFs and T is the fragment flight time. Logarithmic transformation and total differentiation are performed on Equation (1) to establish the relative error propagation function:

(4) $$\begin{array}{c} \frac{dv}{v}=\frac{dL}{L}-\frac{dT}{T} \end{array}$$

Distance and time errors are uncorrelated, and the combined standard uncertainty of velocity is calculated via the root-sum-square (RSS) method:

(5) $$\begin{array}{c} \frac{u_{c}(v)}{v}=\sqrt{{\left(\frac{u(L)}{L}\right)}^{2}+{\left(\frac{u(T)}{T}\right)}^{2}} \end{array}$$

All Type B errors follow a uniform rectangular distribution, with standard uncertainty calculated as $u=\Delta /\sqrt{3}$. Two systematic error sources are considered herein. For distance uncertainty, both fixture assembly tolerance and fragment lateral deflection yield a maximum error of $\Delta L_{1}=\Delta L_{2}=10\;mm$. The individual and combined distance uncertainties are: $u\left(L_{1}\right)=\frac{10}{\sqrt{3}}\approx 5.77\;mm,\;u(L_{2})=\frac{10}{\sqrt{3}}\approx 5.77\;mm$, $u(L)=\sqrt{u(L_{1}{)}^{2}+u(L_{2}{)}^{2}}\approx 8.16\;mm$, $\frac{u(L)}{L}=\frac{8.16}{1000}=0.00816$.

For time uncertainty, errors originate from oscilloscope sampling discretization ($\delta T_{1}=40\;ns$) and 50% voltage-drop threshold identification deviation ($\delta T_{2}=80\;ns$). The corresponding time uncertainties and combined flight time uncertainty are calculated as:$\;u(T_{1})=\frac{40}{\sqrt{3}}\approx 23.09\;ns,u(T_{2})=\frac{80}{\sqrt{3}}\approx 46.19\;ns$, $u\left(T\right)=\sqrt{23.09^{2}+46.19^{2}}\approx 51.67\;ns.$

Given the maximum experimental velocity v=252.72 m/s, the theoretical flight time is $T\approx 3.957\times 10^{6}ns$, corresponding to a negligible relative time uncertainty of $u(T)/T\approx 1.306\times 10^{-5}$. Substituting the error components into Equation (5), the total Type B relative uncertainty is: $\frac{u_{B}(v)}{v}=\sqrt{0.00816^{2}+(1.306\times 10^{-5}{)}^{2}}\approx 0.816\%$.

Clearly, systematic uncertainty is dominated by distance deviation, while time-related error contributes minimally and can be ignored. For Type A statistical uncertainty, six repeated fragment penetration tests were conducted under identical conditions. Statistical analysis of the measured velocity data yields a mean velocity of $\bar{v}\approx 237.05\;m/s$, a single-measurement standard deviation of s(v)≈9.37 m/s, a Type A standard uncertainty of $u_{A}(v)\approx 2.70\;m/s$, and a relative Type A uncertainty of 1.14%.

Type A and Type B uncertainties are independent. The RSS method is used to synthesize the total combined uncertainty. With a coverage factor of k=2 (95% confidence level for engineering measurements), the combined and expanded relative uncertainties are calculated as:$\;\frac{u_{c}(\bar{v})}{\bar{v}}=\sqrt{0.0114^{2}+0.00816^{2}}\approx 1.40\%$, $\frac{U(\bar{v})}{\bar{v}}=2\times 1.40\%=2.80\%$.

The total measurement uncertainty is governed by random experimental dispersion (1.14%) and systematic distance error (0.816%). Random fluctuations stem from launcher instability and fragment morphological differences, whereas systematic errors are caused by assembly tolerance and fragment lateral deflection. Nanosecond-scale time identification errors have no substantial impact on precision. In accordance with GUM criteria, the proposed VMF sensor achieves a combined standard uncertainty of 1.40% and an expanded uncertainty of 2.80% at a 95% confidence level, demonstrating reliable and high-precision velocity measurement performance.

## 4. Conclusions

A high-performance screen-printed VMF with a fast response time and low kinetic energy loss is proposed in this study to address the technical bottlenecks of the long response time of laser-screen targets and the excessive kinetic energy loss of PCB targets. Benefiting from high-conductivity silver grids with a 0.8 mm uniform width and spacing and an ultra-thin 0.175 mm PET substrate, the optimized film structure enables reliable detection of small-diameter fragments with minimal structural interference on fragment flight dynamics.

Circuit simulation and live-fire experimental tests jointly validate the superior transient response performance of the designed VMF. The average measured response time of the VMF is 0.14 μs, which is far lower than the average measured response time of 8.82 μs for the laser-screen target. Penetration experiments further confirm the low-energy-loss advantage of the VMF, with average fragment kinetic energy losses of 1.32 J and 2.09 J for two successive penetration processes.

The differential kinetic energy loss observed in tests indicates minor fragment flight deflection after film penetration, which requires further quantitative investigation and mechanical analysis. Furthermore, all tests in this work are conducted under vertical fragment incidence conditions, which cannot fully replicate the complex oblique penetration scenarios in practical static explosion measurements. Subsequent research will focus on the sensing performance and kinetic energy loss characteristics of the VMF under oblique incidence, so as to further improve its engineering adaptability and practical application value in field blast testing. In the present experiments, the initial flight direction of fragments was perpendicular to the screen-printed film, which differs from the static-detonation measurement scenario. VMF experiments will be carried out at fragment incidence angles of 15°, 30°, 45°, 60°, and 75° to characterize the sensitivity and kinetic energy loss performance of the velocity-measurement film under oblique incidence.

## Figures and Tables

**Table 1:** Response time of laser-screen and VMF.

NO.	$t_{f}$ (μs)	$t_{w}$ (μs)	$\bar{t_{f}}$ (μs)	$\bar{t_{w}}$ (μs)
L1	9.83	12.8	8.82	12.0
L2	7.81	11.1		
F1	0.16	19.2	0.14	21.3
F2	0.12	23.3		
F3	0.13	21.6		
Simulation	0.09	25.2		

**Table 2:** Statistical results of six valid kinetic energy losses.

NO.	$v_{0}$ (m/s)	$v_{1}$ (m/s)	$v_{2}$ (m/s)	$E_{0}$ (J)	$\Delta E_{1}$ (J)	$\Delta E_{2}$ (J)
2	236.41	229.89	225.89	25.06	−1.36	−2.18
3	236.18	229.15	226.30	25.01	−1.47	−2.05
4	237.59	231.00	228.26	25.31	−1.38	−1.95
5	245.94	240.85	237.02	27.12	−1.11	−1.93
6	256.08	249.44	244.68	29.41	−1.51	−2.56
7	257.57	252.72	249.44	29.75	−1.11	−1.85
Mean					−1.32	−2.09

## Data Availability

The raw data supporting the conclusions of this article are included within the article. Further inquiries can be directed to the corresponding author.
